# Biphasic Hormetic-like Effect of Lebecetin, a C-type Lectin of Snake Venom, on Formalin-induced Inflammation in Mice

**DOI:** 10.2174/1570159X22999231207105743

**Published:** 2023-12-08

**Authors:** Carmela Belardo, Jed Jebali, Serena Boccella, Rosmara Infantino, Antimo Fusco, Michela Perrone, Roozbe Bonsale, Iolanda Manzo, Monica Iannotta, Damiana Scuteri, Franca Ferraraccio, Iacopo Panarese, Giovanna Ferrara, Francesca Guida, Livio Luongo, Enza Palazzo, Najet Srairi-Abid, Naziha Marrakchi, Sabatino Maione

**Affiliations:** 1 Department of Experimental Medicine, Pharmacology Division, University of Campania “L. Vanvitelli”, Naples, Italy;; 2 Laboratory of Biomolecules, Venoms and Theranostic Applications, LR20IPT01, Institut Pasteur of Tunis, University of Tunis El Manar, Tunis 1002, Tunisia;; 3 Pharmacotechnology Documentation and Transfer Unit, Preclinical and Translational Pharmacology, Department of Pharmacy, Health and Nutritional Sciences, University of Calabria, Rende, Italy;; 4 Pathology Unit, Department of Mental and Physical Health and Preventive Medicine, University of Campania “L. Vanvitelli”, Naples, Italy

**Keywords:** Snake venom, lebecetin, integrins, formalin, inflammation, pain, mice

## Abstract

**Background:**

Integrins, important extracellular matrix (ECM) receptor proteins, are affected by inflammation and can participate in the maintenance of many painful conditions. Although they are ubiquitous and changeable across all cell types, the roles of these cell adhesion molecules in pathological pain have not been fully explored.

**Objectives:**

We evaluated the effects of the subcutaneous injection of lebecetin, a C-type lectin isolated from *Macrovipera lebetina* snake venom, previously reported to inhibit α5β1 and αv integrin activity, on different components of inflammation induced by the formalin administration in the hind paw of mice.

**Methods:**

The formalin-induced nocifensive behavior, edema, and histopathological changes in the hind paw associated with cytokine, iNOS, and COX2 expression, nociceptive-specific neuron activity, and microglial activation analysis in the spinal cord were evaluated in mice receiving vehicle or lebecetin pretreatment.

**Results:**

Lebecetin inhibited the nocifensive responses in the formalin test, related edema, and cell infiltration in the injected paw in a biphasic, hormetic-like, and dose-dependent way. According to that hormetic trend, a reduction in pro-inflammatory cytokines IL-6, IL-8, and TNF-alpha and upregulation of the anti-inflammatory cytokine IL-10 in the spinal cord were found with the lowest doses of lebecetin. Moreover, COX2 and iNOS expression in serum and spinal cord followed the same biphasic pattern of cytokines. Finally, nociceptive neurons sensitization and activated microglia were normalized in the dorsal horn of the spinal cord by lebecetin.

**Conclusion:**

These findings implicate specific roles of integrins in inflammation and tonic pain, as well as in the related central nervous system sequelae.

## 1 INTRODUCTION

Poisons derived from animals, such as snakes, scorpions, spiders, wasps, frogs, and many others, have gained increasing interest in recent years. Specific structural modifications using biotechnological techniques have made possible a pharmacological application for some of these toxins in various areas of medicine [1, 2]. A modified form of chlorotoxin, a peptide isolated from the scorpion venom *Leiurus quinquestriatus*, has been tested for some forms of astroglioma in humans [3, 4]. Ziconotide, a synthetic analog of a peptide found in the venom of the marine snail *Conus magus*, which blocks presynaptic N-type calcium channels in the spinal cord, is currently approved for severe chronic pain resistant to strong opioids [5]. More impactful was the introduction of exenatide, a synthetic analog of exendin-4 isolated from the saliva of the Gila monstrous lizard, for the treatment of diabetes mellitus [6, 7]. Therefore, despite the high toxicological properties of venoms, many peptides derived from animal venom paradoxically show therapeutic potential as thrombolytic, antiplatelet, and antiangiogenic agents , associated with anti-metastatic and anti-proliferative effects [8].

The venom of the *Macrovipera lebetina* is an amalgam of different proteins, including proteases, phosphodiesterases, phosphatases, phospholipase A2, disintegrins, and C-type lectin-like proteins [8-14]. Among the C-type lectin-like proteins from *Macrovipera lebetina*, lebectin and lebecin, showed a powerful inhibition of platelet aggregation, cell adhesion, migration, invasion, and proliferation by inhibiting the α5β1 and αv integrins without inhibition of the collagen α2β1 receptor [15, 16]. Another C-type lectin-like protein isolated from the same venom lebecetin displayed anti-integrin activity by inhibiting the α5β1 and αv integrins [15, 17]. Lebecetin showed some pharmacological effects, including i) inhibition of thrombin- and collagen-induced platelet aggregation [18, 19], ii) inhibition of adhesion, migration, and proliferation of tumor and microvascular endothelial cells [17], and iii) inhibition of choroidal and retinal neovascularization [20]. Lebecetin also shaped the inflammatory response *in vitro* by inhibiting the pro-inflammatory cytokines TNF-α, IL-6, and IL-8 or stimulating the expression of the anti-inflammatory cytokine IL-10 following exposure to the bacterial lipopolysaccharide, LPS, in human THP-1-derived macrophages. This effect was associated with an inhibition of ERK1/2, p38, AKT kinases, NF-κB, and αvβ3 integrin expression [21]. Despite various biological activities, including a potential anti-inflammatory effect of lebecetin, and evidence demonstrating that integrins have a role in inflammatory or neuropathic pain [22], the analgesic and anti-inflammatory effect of lebecetin *in vivo* has not yet been investigated. Injection of formalin into the hind paw of rodents induces inflammatory pain, which allows monitoring of both, acute and tonic pain responses and the underlying inflammatory, neurogenic, and central mechanisms of nociception [23-25]. On this basis, the current study aims to investigate the effect of lebecetin on nocifensive responses, paw edema, and histopathological features induced by the administration of formalin into the hind paw. The effects of lebecetin on formalin-induced neuronal sensitization, microglia changes, iNOS, COX2, and pro-inflammatory/anti-inflammatory cytokines expression in the dorsal horn of the spinal cord were also investigated. Hopefully, these data will highlight the potential therapeutic application of C-type lectin-like biomolecules in the management of inflammatory lesions with an important painful component involved in the development and progression of several diseases.

## 2 MATERIALS AND METHODS

### 2.1 Animals

Adult male C57/BL6J mice (Envigo, Milan, Italy), weighing 22-25 g, were housed in 3 per cage under controlled illumination (12:12 hr light-dark cycle; light on 6.00 a.m.) and standard environmental conditions (ambient temperature 20-22°C, humidity 55-60%) for at least 1 week before the commencement of experiments. Mouse chow and tap water were available ad libitum. The experimental tests were carried out during the light phase. The experimental procedures were approved by the Animal Ethics Committee of the University of Campania “L. Vanvitelli” (Naples, Italy). Animal care complied with Italian (D.L. 116/92) and EEC (O.J. of E.C. L358/1 18/12/86) regulations on the protection of laboratory animals. All efforts were made to minimize animal suffering and reduce the number of animals used.

### 2.2 Formalin Test

Each mouse was randomly assigned to one of the experimental groups, placed in a plastic cage, and allowed to move freely for 30 min. A mirror was placed at a 45° angle under the cage to allow a full view of the hind paws. Vehicle (PBS, 1%, 30 μl) or different doses of lebecetin (0.15, 0.32, 0.64, and 1.31 nmol in 30 μl) were subcutaneously administered 10 min before injecting formalin (1.25%, 30 μl) into the dorsal surface of the hind-paw. Lifting, licking, shaking, and flinching of the injected paw were recorded as nocifensive responses. Recording of nocifensive behavior scored in 5-min blocks was commenced immediately after the injection of formalin and was continued for 60 min. Groups of 7-10 mice per treatment were used, with each animal used for one treatment only. Results were expressed as mean ± S.E.M. of the total time spent in nocifensive response in min.

### 2.3 Determination of Edema Formation

The hind paw edema in mice was measured before (0 h) and at 60 minutes after formalin injection into the dorsal surface of the hind paw using an analogic caliper (Stainless Steel Digital Caliper, Bay, Gaithersburg, MD, USA). Data were recorded and expressed as mean ± S.E.M. of paw thickness in mm to the nearest ± 0.01 mm.

### 2.4 *In Vivo* Single-unit Electrophysiological Recordings of Spinal Nociceptive-specific Neurons

For *in vivo* single unit extracellular recording, mice were anesthetized with avertin (1.25%). After tracheal cannulation, a catheter was placed into the right external jugular vein to allow a continuous further infusion of propofol (5-10 mg/kg/h, i.v.). Spinal cord L4-L6 segments were exposed medially by laminectomy, near the dorsal root entry zone, up to a depth of 1 mm. Mice were then secured in a stereotaxic apparatus (David Kopf Instruments, Tujunga, CA, United States) supported by clamps attached to the vertebral processes on either side of the exposure site. An elliptical rubber ring (about 3 mm × 5 mm) was tightly sealed with silicone gel onto the surface of the spinal cord. This ring formed a depression that provided access to spinal neurons receiving input from the ipsilateral paw, where formalin, vehicle, or solutions containing different doses of lebecetin were injected. The exposed area of the spinal cord was framed with agar and then filled with mineral oil. Body temperature was maintained at 37°C with a temperature-controlled heating pad. A glass-insulated tungsten filament electrode (3-5 MΩ; FHC Frederick Haer& Co., Bowdoin, ME, United States) was used to record the single-unit extracellular activity of dorsal horn nociceptive-specific (NS) neurons. NS neurons were defined as those neurons that respond only to high-intensity (noxious) stimulation. To confirm NS neuron response patterns, each neuron was characterized while applying mechanical stimulation to the ipsilateral hind paw using a von Frey filament with 97.8 mN bending force (noxious stimulation) for 2 s until it buckled slightly. Only neurons that responded specifically to the noxious hind paw stimulation without responding to stimulation of the surrounding tissue were considered. The recorded signals were amplified and displayed on a digital storage oscilloscope to ensure that the unit under study was unambiguously discriminated throughout the experiment. Signals were also fed into a window discriminator, whose output was processed by an interface CED 1401 (Cambridge Electronic Design Ltd., Milton, United Kingdom) connected to a Pentium III PC. Spike2 software (CED, version 4) was used to create peristimulus rate histograms online and to store and analyze digital records of single-unit activity offline. Configuration, shape, and height of the recorded action potentials were monitored and recorded continuously using a window discriminator and the Spike2 software for online and offline analysis. This study included only neurons whose peak configuration remained constant and discriminated against background activity, thus allowing the activity of a single neuron and the same neuron to be analyzed throughout the experiment. The spontaneous and noxious-evoked neuronal activity was expressed as spikes/s (Hz) for the spontaneous activity and frequency of excitation and in seconds (s) for the duration of excitation. At the end of the experiment, each animal was sacrificed with a lethal dose of urethane.

### 2.5 Immunoassays (ELISA)

Enzyme-linked immunosorbent assays (ELISAs) were performed to analyze the levels of proinflammatory and antiinflammatory cytokines (IL1β, IL-6, IL-8, IL-10, and TNF-α) in the spinal cord and COX2 and iNOS enzymes in the spinal cord and serum (Elabscience, Houston, ID, USA). Tissue samples of the spinal cord were weighed and homogenized in PBS 0.01M pH = 7.4 (tissue weight (g): PBS ml = 1:9). The homogenates were centrifuged for 5-10 min at 5000 × g at 4°C to get the supernatant. The serum was also collected and left to clot overnight at 2-8°C before centrifugation for 20 min at 1000×g at 2-8°C. The supernatant was collected to carry out the assay.

### 2.6 Immunofluorescence

Under anesthesia, mice were transcardially perfused with a saline solution followed by 4% paraformaldehyde in 0.1 M phosphate buffer. The lumbar spinal cord was excised, post-fixed for 4 h in the perfusion fixative, cryoprotected for 72 h in 20% sucrose in 0.1 M phosphate buffer and frozen in O.C.T. embedding compound. Transverse sections (20 micrometers) were cut using a cryostat and thaw-mounted onto glass slides. Slides were incubated overnight with primary antibody solutions for the microglial cell marker Iba-1 (rabbit anti-ionized calcium-binding adapter molecule 1; 1:1000; Wako Chemicals, Germany). Following incubation, sections were washed and incubated for 3 h with a secondary antibody solution (goat anti-rabbit, IgG-conjugated Alexa FluorTM 488; 1:1000; Molecular Probes, USA). Slides were washed, cover-slipped with Vectashield mounting medium (Vector Laboratories, USA) and visualized under a Leica fluorescence microscope.

The numbers of profiles positive for each marker were determined within a box measuring 104 um^2^ in the lateral, central, and medial areas of the dorsal horn spinal cord sections. Eight L5 spinal sections were evaluated from each of the three mice per group. An average value obtained by combining the values of the lateral, central, and medial areas of both dorsal and ventral horns was considered. Mean values for three mice per group were then compared using one-way ANOVA followed by Tukey’s post hoc test. *P* < 0.05 was set as the level of statistical significance.

### 2.7 Histopathology Examination

For histological examination, biopsies of the hind paws were taken 60 min following the subcutaneous administration of formalin. All tissue samples were fixed in 10% neutral buffered formalin solution, embedded in paraffin wax, cut into 10 μm thick sections, and stained with hematoxylin-eosin. All samples were photographed with a Canon EOS700 camera connected to an optical microscope.

### 2.8 Drugs

Venom was collected from *M. lebetina* snake in the serpentarium of the Pasteur Institute of Tunisia. Lebecetin was purified to homogeneity from *M. lebetina* venom by gel filtration on a Sephadex G75 column followed by ion exchange chromatography on a Mono S column and characterized as previously described [18]. The lyophilized lebecetin was dissolved in PBS at a concentration of 1.31 nmol (stock solution) and subsequently diluted at 0.15, 0.32, 0.64, and 1.31 nmol in 30 microliters. A 10% formalin solution was purchased from Sigma-Aldrich, St Louis, MO, USA and diluted at 1.25% in 30 ml.

### 2.9 Statistics

Data were represented as mean ± SEM. Behavioral data were analyzed by two-way ANOVA followed by Dunnet's post-hoc test. Paw thickness and *in vitro* data were analyzed by one-way ANOVA followed by Dunnett’s post-hoc test. Iba-1 immunoreactivity was analyzed by one-way ANOVA followed by Tukey’s post hoc test. Electrophysiological recording data were analyzed by two-way ANOVA followed by Tukey’s post-hoc test. *P* values < 0.05 were considered statistically significant. Statistical analysis was carried out using Prism/Graphpad (GraphPad Software, Inc.) software.

## 3 RESULTS

### 3.1 Effect of Different Doses of Lebecetin on Formalin-induced Nocifensive Behavior

**Fig. (1):**
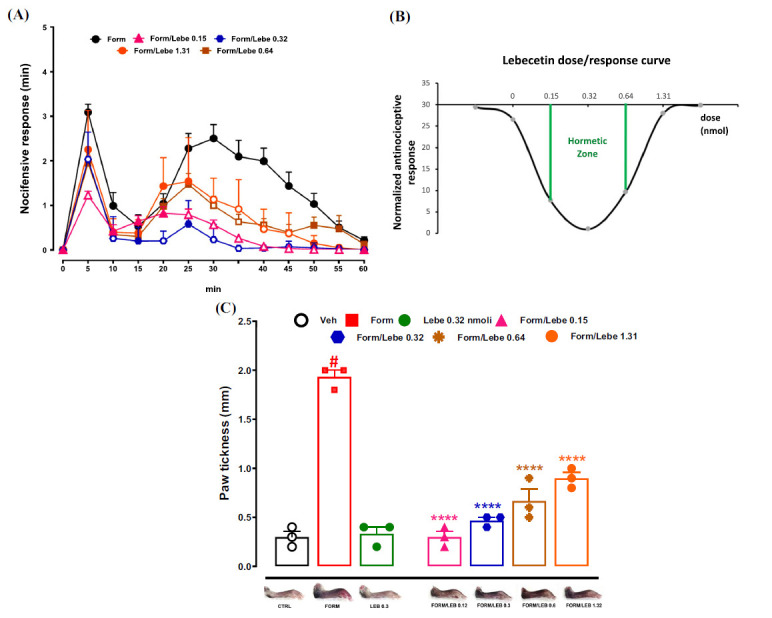
(**A**) shows the effect of vehicle (Veh, PBS 1%, 30 μl) or different doses of lebecetin (Lebe, 0.15, 0.32, 0.64, and 1.31 nmol in 30 μl) administered in the dorsal surface of the hind paw 10 min before formalin (Form, 1.25%, 30 μl) on the nocifensive response expressed in min. (**B**) shows the dose-response curve (hormesis) of Lebecetin built on the pro/antinociceptive response in formalin-injected mice. The responses obtained at different nmol doses were normalized to the maximum antinociceptive effect (0.32 nmol). (**C**) shows the effect of vehicle (Veh, PBS 1%, (30 μl), formalin (Form, 1.25%, 30 μl), lebecetin (Lebe, 0.32 nmol, 30 μl), or different doses of lebecetin (Lebe, 0.15, 0.32, 0.64, and 1.31 in 30 μl)) administered in the dorsal surface of the hind paw 10 min before formalin (Form, 1.25%, 30 μl) on paw thickness expressed in mm and photographs showing hind paw edema after subcutaneous injection of formalin (Form) and ameliorative effect after pretreatment (10 min before formalin) with different lebecetin doses (Lebe, 0.15, 0.32, 0.64, and 1.31 nmol). Each time point represents the mean ± SEM of 5-10 (**A**) or 3 (**C**) mice per group. *p* < 0.05 was considered statistically significant, two-way ANOVA followed by Dunnet's post-hoc test (**A**) and one-way ANOVA followed by Dunnett’s post-hoc test (**C**). Filled symbols indicate statistically significant time points in panel A. # indicates *p* < 0.05 *vs.* vehicle-injected mice, and ****p < 0.001 *vs.* formalin-injected mice in panel C.

In mice that received the subcutaneous injection of vehicle (veh, PBS 1%, 30 μl) 10 min before formalin (Form, 1.25%, 30 μl) in the dorsal surface of the hind paw, the first phase of noxious behavior lasting for 0-10 min was observed immediately after the injection of formalin. The nocifensive behavior in the first phase was 3.08 ± 0.18 min. This phase was followed by a second phase of nociceptive behavior, reflecting the development of nociceptive sensitization in the dorsal horn of the spinal cord [24, 26]. The nocifensive behavior in the second phase was 2.09 ± 0.63 min at 30 min after formalin administration. Subcutaneous administration of the lowest doses of lebecetin (Lebe, 0.15 nmol and 0.32 nmol in 30 μl) 10 min before the formalin administration reduced the nocifensive behavior in the first phase (1.22 ± 0.09 min and 2.03 ± 0. 19 min, respectively). Such doses of lebecetin (0.15 nmol and 0.32 nmol in 30 μl) also reduced the second phase of nocifensive behavior (0.56 ± 0.10 min and 0.23 ± 0.1 min, respectively). Higher doses of lebecetin (0.64 nmol and 1.31 nmol in 30 μl) did not modify the nocifensive behavior in the first phase (1.94 ± 0.32 min and 2.24 ± 0.29 min, respectively), while they reduced the nocifensive behavior in the second phase of the formalin test (0.63 ± 0.17 min and 0.91 ± 0.22 min, respectively) compared to vehicle-injected mice (Fig. **1A**). The dose-response curve of different doses of lebecetin in formalin-injected mice is presented in Fig. (**1B**).

### 3.2 Effect of Different Doses of Lebecetin on Formalin-induced Paw Edema

In mice that received the subcutaneous injection of vehicle (Veh, PBS 1% in 30 μl) in the dorsal surface of the hind paw, the paw thickness was 0.3 ± 0.05 mm. The administration of formalin (1.25%, 30 μl) increased the paw thickness (1.93 ± 0.06 mm) compared to the paw thickness of mice that received the vehicle. The administration of the different doses of lebecetin (Lebe, 0.15, 0.32, 0.64, and 1.31 nmol in 30 μl) 10 min before the formalin reduced the paw thickness (0.3 ± 0.05 mm, 0.46 ± 0.03 mm, 0.66 ± 0.12 min, and 0.9 ± 0.05 mm, respectively) compared to the paw thickness of mice that received the administration of vehicle 10 min before the administration of formalin (Fig. **1C**).

### 3.3 Effect of Lebecetin on Spinal NS Neuron Activity

**Fig. (2):**
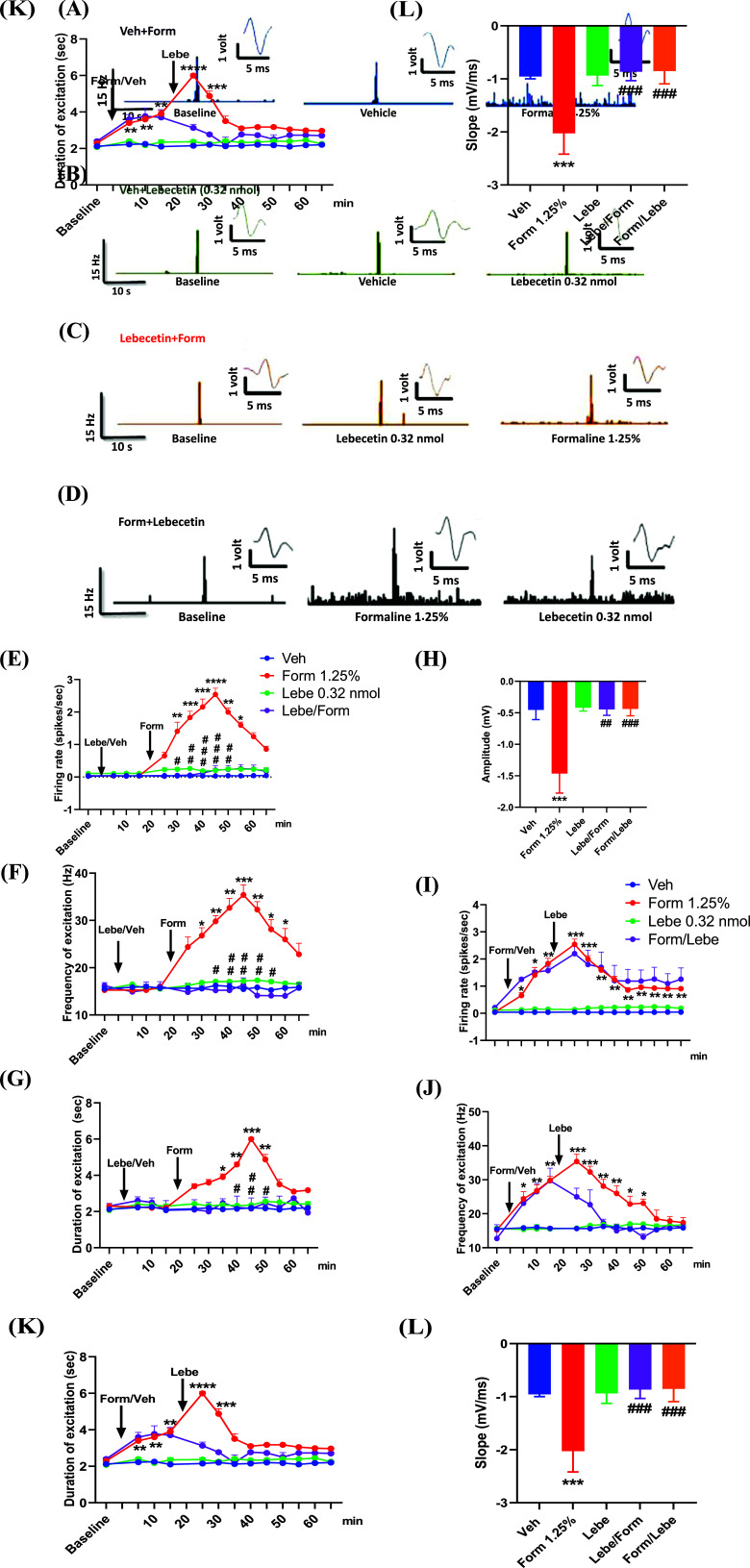
Representative ratematers showing the spontaneous and noxious-evoked activity of spinal NS neurons before (baseline) and after the subcutaneous administration of vehicle (PBS 1%) 10 min before formalin (Form, 1.25%, 30 μl) (**A**) or lebecetin (0.32 nmol, 30 μl) (**B**) in the dorsal surface of the hind paw. (**C**) shows representative ratematers showing the spontaneous (baseline) and noxious-evoked activity of spinal NS neurons before and after the subcutaneous administration of lebecetin (0.32 nmol, 30 μl) 10 min before formalin (Form, 1.25%, 30 μl) whereas (**D**) shows representative ratematers showing the spontaneous and noxious-evoked activity of spinal NS neurons before (baseline), and after the subcutaneous administration of formalin (Form, 1.25%, 30 μl) 10 min before lebecetin (0.32 nmol, 30 μl). Effect of the subcutaneous administration of vehicle (Veh, PBS 1%, 30 μl), formalin (Form, 1.25%, 30 μl), lebecetin (Lebe, 0.32 nmol, 30 μl) or lebecetin (0.32 nmol, 30 μl) administered 10 min before formalin on the firing rate (**E**), frequency of excitation (**F**), and duration of excitation (**G**). Effect of the administration of vehicle (Veh, PBS 1%, 30 μl), formalin (Form, 1.25%, 30 μl), lebecetin (Lebe, 0.32 nmol, 30 μl), lebecetin administered 10 min before formalin (Lebe/Form), and lebecetin administered 10 min after formalin (Form/Lebe) on the amplitude (mV) of spinal NS neurons (**H**). The effect of the subcutaneous administration of vehicle (Veh, PBS 1%), formalin (Form, 1.25%, 30 μl) or lebecetin (Leb, 0.32 nmol, 30 μl) on the firing rate (**I**), frequency of excitation (**J**), and duration of excitation (**K**) was also evaluated when lebecetin (0.32 nmol, 30 μl) was administered 10 min after formalin. Effect of the administration of vehicle (Veh, PBS 1%, 30 μl), formalin (Form, 1.25%, 30 μl), lebecetin (Lebe, 0.32 nmol, 30 μl), lebecetin administered 10 min before formalin (Lebe/Form), and lebecetin administered 10 min after formalin (Form/Lebe) on the slope (mV/ms) (**L**) of spinal NS neurons. Each point represents the mean ± S.E.M. ***indicates *p* < 0.005 *vs.* vehicle, ^##^indicates *p* < 0.01, and ^###^*p* < 0.005 *vs.* form (two-way ANOVA followed by Tukey’s post-hoc test).

The administration of the vehicle into the hind paw did not affect the spontaneous activity (0.035 ± 0.03 spikes/s), frequency of excitation (15.46 ± 0.44 spikes/s), and the duration of excitation (2.20 ± 0.071 s) of spinal NS neurons compared to respective pre-administration values (0.04 ± 0.005 spikes/s, 15.53 ± 0.36 spikes/s, and 2.12 ± 0.075 s) (Fig. **2**). The administration of formalin (1.25%, 30 ul) into the dorsal surface of the hind paw increased the spontaneous activity (2.54 ± 0.19 spikes/s, 25 minutes after the injection of formalin), frequency of excitation (35.37 ± 2.16 spikes/s, 25 minutes after the injection of formalin), and the duration of excitation (6.00 ± 0.091 s, 25 minutes after the injection of formalin) of spinal NS neurons as compared to baseline (0.042 ± 0.004 spikes/s, *p* = 0.031; 15.26 ± 0.32 spikes/s, *p* = 0.0065 and 2.3 ± 0.00 s, *p* < 0.0001 for the spontaneous activity, frequency of excitation, and duration of excitation, respectively) (Fig. **2**). Administration of lebecetin (0.32 nmol, 30 μl) alone into the dorsal surface of the hind paw did not affect the spontaneous activity (0.22 ± 0.057 spike/s, *p* = 0.98), frequency of excitation (16.22 ± 0.32 spikes/s, *p* = 0.99), and duration of excitation (2.42 ± 0.04 s, *p* = 0.89) of spinal NS neurons 25 minutes after the administration of lebecetin as compared to the NS neuron activity of mice treated with vehicle (Fig. **2**). When lebecetin (0.32 nmol, 30 μl) was administered 10 min before formalin, it significantly prevented the formalin-induced spinal hyperexcitability. Indeed 25 min after the administration of formalin, the spontaneous activity of spinal NS neurons was 0.21 ± 0.13 spike/s (*p* < 0.0001), the frequency of excitation was 16.37 ± 1.48 spikes/s (*p* < 0.0001), and the duration of excitation was 0.61 spikes/s (*p* <0.05), as compared to the NS neuron activity in mice receiving only formalin (Fig. **2C**). Two-way ANOVA followed by Tukey’s post-hoc test revealed a significant effect of time (F_(12, 143)_ = 32.33, *p* < 0.0001), treatment (F_(3, 143)_ = 442.5, *p* < 0.0001) and a significant interaction time X treatment (F_(36, 143)_ = 25.58, *p* < 0.0001), in the preventive effect of lebecetin. This effect was partially lost when lebecetin was administered 10 min after formalin administration. Indeed, it only slightly counteracted the sensitization of NS neurons found in the group of mice receiving formalin alone (Fig. **2**). However, two-way ANOVA followed by Tukey’s post-hoc test revealed a significant effect of time (F_(12, 143)_ = 7.265, *p* < 0.0001), treatment (F_(3, 143)_ = 144.5, *p* < 0.0001) and a significant interaction time X treatment (F_(36, 143)_ = 2.72, *p* < 0.0001), when lebecetin was injected after the formalin application. Finally, formalin-induced spinal hyperexcitability corresponded to a significant increase in amplitude (-1.46 ± 0.31 mV, *p* < 0.001) and slope (-2 ± 0.39 mV/ms, *p* < 0.001) of extracellular action potentials (eAPs), as compared to the vehicle-injected group of mice (-0.45 ± 0.15 mV for amplitude, and -0.95 ± 0.04 mV/ms for slope) (Fig. **2**). The administration of lebecetin both before and after the injection of formalin was able to normalize the amplitude (-0.44 ± 0.09 mV, *p* < 0.001 for Lebe + Form, and -0.45 ± 0.1 mV, *p* < 0.001 for Form + Lebe) and slope (-0.86 ± 0.16 mV/ms, *p* < 0.001 for Lebe + Form, and -0.85 ± 0.24 mV/ms, *p* < 0.001 for Form + Lebe) (Fig. **2**).

### 3.4 Effect of Lebecetin on the Expression Levels of Cytokines in the Spinal Cord

The levels of IL-1β, IL-4, IL-6, IL-8, IL-10, and TNF-α in the spinal cord and those of iNOS and COX2 in the serum and spinal cord were measured by ELISA assay.

**Fig. (3):**
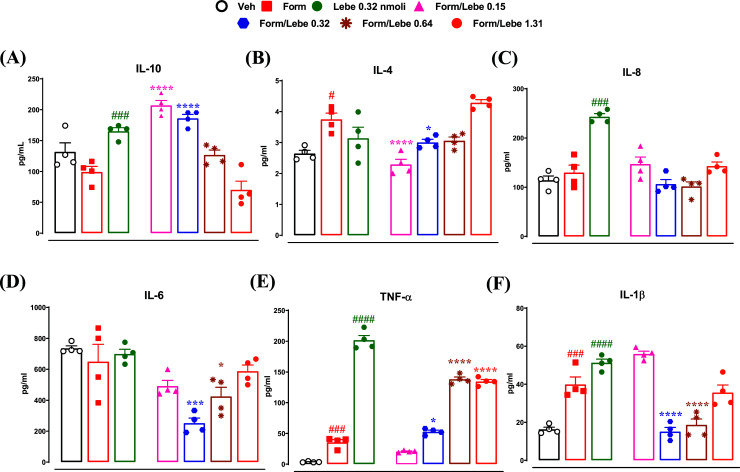
Effect of vehicle (Veh, PBS 1%, 30 μl), formalin (Form, 1.25%, 30 μl), lebecetin (Lebe, 0.32 nmol, 30 μl) or different doses of lebecetin (Lebe, 0.15, 0.32, 0.64, and 1.31 nmol in 30 μl) administered in the dorsal surface of the hind paw 10 min before formalin on cytokines levels expressed in pg/ml in the spinal cord. In particular, the effects of the above treatments on IL-10 (**A**), IL-4 (**B**), IL-8 (**C**), IL-6 (**D**), TNF-α (**E**), and IL-1β (**F**) are shown. Each histogram represents the mean ± SEM of 4 mice per group. *p* < 0.05 was considered statistically significant (one-way ANOVA followed by Dunnett’s post-hoc test). ^#^indicates *p* < 0.05, ^###^*p* < 0.005, and ^####^*p* < 0.001 *vs*. vehicle-injected mice. *indicates *p* < 0.05, ***p < 0.005, and ****p < 0.001 *vs.* formalin-injected mice.

A downward trend, even if not significant, in the expression levels of the anti-inflammatory cytokine IL-10 (99.21 ± 8.95, F(1,9) = 5.085, *p* = 0.0506) was found in the spinal cord of mice receiving the peripheral administration of formalin compared to the levels of IL-10 found in control mice (131.8 ± 14.54). An increasing trend, even if not significant, of IL-10, was found (165.0 ± 5.83, *p* = 0.056) in the spinal cord of mice receiving the lebecetin (0.32 nmol) into the hind paw. The levels of IL-10 in the spinal cord increased in mice treated with low doses of lebecetin (0.15 and 0.32 nmol) 10 min before the administration of formalin (207.1 ± 7.85, *p* < 0.0001 and 186.1 ± 6.21, *p* < 0.0001, respectively). The pretreatment with lebecetin at the doses of 0.64 and 1.31 nmol did not change the IL-10 levels in the spinal cord (126.8 ± 7.47 and 79.26 ± 13.99, respectively) (Fig. **3A**).

The levels of the immunomodulatory cytokine, IL-4, were increased (3.75 ± 0.19, F(6,21) = 19.30, *p* < 0.0001) in the spinal cord of mice receiving the peripheral administration of formalin compared to control mice (2.64 ± 0.10). The levels of IL-4 did not change (3.14 ± 0.35) in the spinal cord of mice receiving the administration of lebecetin (0.32 nmol) into the hind paw. The levels of IL-4 in the spinal cord decreased (2.29 ± 0.16, *p* < 0.0001 and 3.0 ± 0.09, *p* < 0.04, respectively) in mice treated with low doses of lebecetin (0.15 and 0.32 nmol) 10 min before the administration of formalin. Also the dose of lebecetin of 0.64 nmol (30 μl) 10 min before formalin decreased IL-4 levels in the spinal cord (3.05 ± 0.12). The pretreatment with lebecetin 1.31 nmol did not reduce the IL-4 levels in the spinal cord (4.29 ± 1.99) (Fig. **3B**).

The levels of IL-8, a chemotactic factor released by macrophages, did not change (129.8 ± 15.11) in the spinal cord of mice receiving the peripheral administration of formalin compared to control mice (114 ± 8.56). The levels of IL-8 were increased (243.4 ± 6.16) in the spinal cord of mice receiving the lebecetin (0.32 nmol) into the hind paw. The pretreatment with different doses of lebecetin (0.15, 0.32, 0.64, and 1.31 nmol) 10 min before the peripheral administration of formalin did not change the levels of IL-8 in the spinal cord (Fig. **3C**).

The expression levels of IL-6 did not change (649.9 ± 15.96, F(6,21) = 9.74, *p* = 0.7352) in the spinal cord of mice receiving the peripheral administration of formalin compared to control mice (735.7 ± 15.96). The levels of IL-6 did not change (699.9 ± 24.96) also in the spinal cord of mice receiving the administration of lebecetin (0.32 nmol) into the hind paw. The pretreatment with lebecetin at the doses of 0.32 and 0.64 nmol, 10 min before the peripheral administration of formalin, decreased the levels of IL-6 (252 ± 32.52 and 424.9 ± 58.73, respectively) in the spinal cord (Fig. **3D**). The doses of 0.15 and 1.31 nmol of lebecetin administered 10 min before the peripheral administration of formalin, were instead unable to change the IL-6 levels in the spinal cord (Fig. **3D**).

The expression levels of the TNF-α increased (35.55 ± 4.34, F(6,14) = 289.8, *p* < 0.0001) in the spinal cord of mice treated with formalin compared to the levels of TNF-α in control mice (3.63 ± 0.464). The levels of TNF-α increased (201.6 ± 7.63) in the spinal cord of mice receiving the lebecetin (0.32 nmol) into the hind paw. The pretreatment with lebecetin (0.15 and 0.32 nmol) administered 10 min before the peripheral administration of formalin, did not change the TNF-α levels (26.40 ± 4.75 and 52.81 ± 2.52), while the levels of TNF-α in the spinal cord of mice receiving lebecetin at doses of 0.64 and 1.31 nmol were found greatly increased (138.1 ± 3.71 and 134.4 ± 3.20, *p* < 0.0001) (Fig. **3E**).

The levels of the immunomodulatory cytokine IL-1β increased (39.92 ± 3.88, F(6,21) = 39.06; *p* < 0.0001) in the spinal cord of mice receiving the peripheral administration of formalin compared to control mice (16.34 ± 1.10). The levels of IL-1β increased (51.32 ± 1.87, *p* = 0.03) in the spinal cord of mice receiving the administration of lebecetin (0.32 nmol) into the hind paw. The levels of IL-1β were decreased by the pretreatment with the intermediate doses of lebecetin (0.32 and 0.64 nmol) 10 min before the peripheral administration of formalin (15.17 ± 2.13 and 18.67 ± 3.09, *p* < 0.0001). The pretreatment with the doses of 0.15 and 1.31 nmol of lebecetin, 10 min before the peripheral administration of formalin, did not change the IL-1β levels in the spinal cord (Fig. **3F**).

### 3.5 Effect of Lebecetin on the Expression Levels of COX-2 and iNOS in the Serum and Spinal Cord

**Fig. (4):**
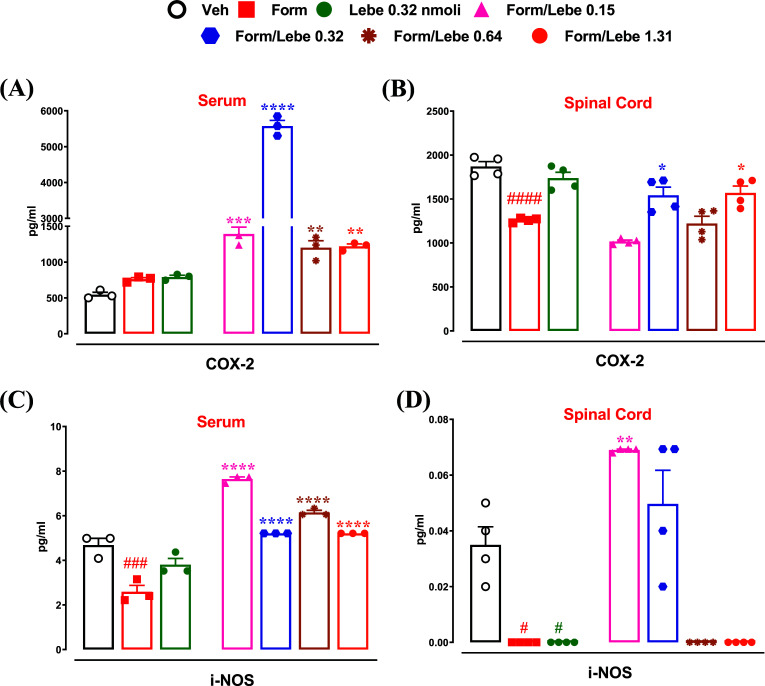
Effect of vehicle (Veh, PBS 1%, 30 μl), formalin (Form, 1.25%, 30 μl), lebecetin (Lebe, 0.32 nmol, 30 μl) or different doses of lebecetin (Lebe, 0.15, 0.32, 0.64, and 1.31 nmol in 30 μl) administered in the dorsal surface of the hind paw 10 min before formalin on COX-2 (**A** and **B**) and i-NOS (**C** and **D**) levels expressed in pg/ml in the serum (**A** and **C**) and spinal cord (**B** and **D**). Each histogram represents the mean ± SEM of 3-4 mice per group. *P* < 0.05 was considered statistically significant (one-way ANOVA followed by Dunnett’s post-hoc test). ^#^indicates *p* < 0.05, ^###^*p* < 0.005 and ^####^*p* < 0.001 *vs.* vehicle-injected mice. *indicates *p* < 0.05, **p < 0.01, ***p < 0.005, and ****p < 0.001 *vs.* formalin-injected mice.

The peripheral administration of formalin did not change the levels of COX-2 in the serum (762.1 ± 21.85) (Fig. **4A**), while it decreased the levels of COX-2 in the spinal cord (1261 ± 12.03, F(6,21) = 22.01; *p* < 0.0001) (Fig. **4B**) compared to the levels found in the serum (546.9 ± 32.78) and spinal cord (1870 ± 59.93,) of control mice. The administration of lebecetin (0.32 nmol) alone did not change the levels of COX-2 in both the serum (792 ± 24.05) and spinal cord (1738 ± 66.91). The pretreatment with lebecetin (0.15, 0.32, 0.64, and 1.31 nmol) 10 min before the administration of formalin increased the expression levels of COX-2 in the serum (1392 ± 94.91, 5574 ± 157.5, 1201 ± 96.21, and 1220 ± 32.78, respectively) (Fig. **4A**). Only the doses of 0.32 and 1.31 nmol of lebecetin increased the COX-2 expression levels in the spinal cord (1542 ± 92.76 and 1570 ± 78.42, respectively) (Fig. **4B**).

The peripheral administration of formalin decreased the levels of i-NOS in the serum (2.59 ± 0.28 F(6,21) = 68.45, *p* < 0.0001) (Fig. **4C**) and spinal cord (0.035 ± 0.006 F(6,21) = 18.65, *p* < 0.0001) (Fig. **4D**) compared to the i-NOS expression levels in the serum (4.68 ± 0.29) and spinal cord (0.035 ± 0.006) of control mice. The administration of lebecetin (0.32 nmol) alone did not change the levels of i-NOS in the serum (3.80 ± 0.28) (Fig. **4C**) while it decreased the i-NOS levels in the spinal cord (0 ± 0, *p* < 0.0001) (Fig. **4D**). The pretreatment with lebecetin (0.15, 0.32, 0.64, and 1.31 nmol) 10 min before the peripheral administration of formalin increased the i-NOS expression levels in the serum (7.64 ± 0.09, 5.21 ± 0.0, ± 0.614 ± 0.09, and 5.21 ± 0.0 respectively) (Fig. **4C**). Only the doses of 0.15 and 0.32 nmol of lebecetin administered 10 min before formalin increased the i-NOS levels in the spinal cord (0.06 ± 0 and 0.049 ± 0, respectively), while the doses of 0.64 and 1.31 were ineffective (Fig. **4D**).

### 3.6 Effect of Lebecetin on Spinal Microglia Activation

**Fig. (5):**
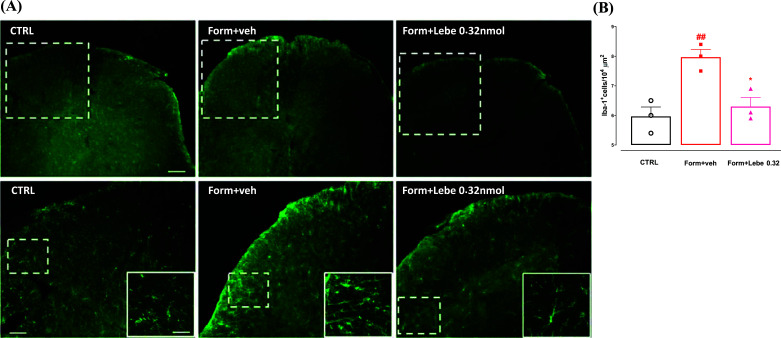
Effect of vehicle (Veh, PBS 1%, 30 μl) or lebecetin (Lebe, 0.32 nmol, 30 μl) administered in the dorsal surface of the hind paw 10 min before formalin (Form, 1.25%, 30 μl) on Iba-1 immunoreactivity (Iba-1-ir) in the dorsal horns of the spinal cord (**A**). Quantitative analysis of spinal cord sections shows increased numbers of Iba-1 positive microglia in the dorsal horn of spinal cord in mice receiving vehicle 10 min before the administration of formalin compared to control mice. Lebecetin (0.32 nmol, 30 μl), administered 10 min before formalin into the dorsal surface of the hind paw significantly reduced the number of the Iba-1 positive profiles (**B**). Data represent mean ± S.E.M., n = 3 mice per group. *p* < 0.05 was considered statistically significant. ^##^indicates *p* < 0.01 *versus* control mice, whereas ° indicates *p* < 0.05 *versus* formalin-injected mice, one-way ANOVA followed by Tukey’s post hoc test. Scale bars = 500, 100, 50 um in the low magnification, medium magnification and inset, respectively.

The microglia component plays a pivotal role in the second phase of formalin-induced nocifensive behaviour. Indeed, it has been recently shown that spinal microglia contribute to sustained inflammatory pain *via* amplifying neuronal activity [27]. We found a significantly increased number of Iba1-positive microglia profiles in the dorsal horn of the spinal cord in the formalin-injected mice compared to the vehicle-injected control group (Fig. **5**). Interestingly, the intra-paw injection of lebecetin (0.32 nmol), at the same dose that reduced the second phase of formalin-associated painful behavior, reduced the number of microglia cells.

### 3.7 Histopathological Changes Induced by Lebecetin in Formalin-associated Paw Inflammation

**Fig. (6):**
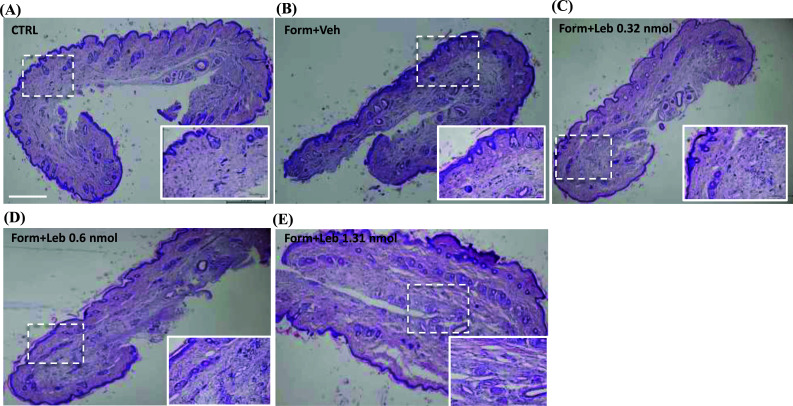
Representative photographs from the skin show the protective effect of lebecetin against formalin-induced inflammation and edema in dorsal paw skin (H&E stain) in mice. (**a**) Control; (**b**) formalin + vehicle; (**c**) formalin + lebecetin 0.32 nmol; (**d**) formalin +lebecetin 0.64 nmol; (**e**) formalin + lebecetin 1.31 nmol.

The skin biopsies of the dorsal paw showed marked cellular infiltration in the connective tissue with edema in both the epidermis and dermis. Formalin induced the appearance of diffuse spongy tissue swelling within the subcutaneously injected areas. The vascular diameter enlargement was observed which could be suggestive of a diffuse hyperemia around the vessels in the dermis. Moreover, lympho-monocytic infiltration was diffusely found in the epidermis and dermis. The biopsies from mice treated with lebecetin showed a partial reduction in the inflammatory response only with the lowest dose (0.32 nmol). The highest doses worsened the formalin-induced inflammatory state as inflammatory cells were more diffusely in number or near the vascular areas as compared to the formalin-injected group. Yet, lebecetin at the higher doses enhanced the subcutaneous tissue swelling and vascular diameter enlargement was larger as compared to the formalin group (Fig. **6**).

## 4 DISCUSSION

Pain and inflammation are still not treated effectively and often remain unsolved clinical problems, especially after chronicization. The lack of corrective drugs to address these clinical issues could be directly related to the fact that the underlying mechanisms of chronic pain and inflammation are only partially understood, and the reported information is often conflicting [28]. The sensitization of the neurons involved in the transmission of nociceptive inputs is, in many cases, associated with the inflammatory cascade and the peripheral neurochemical alterations linked to the lesion of the nerve fibers. The formalin test, through the peripheral injection of a formalin-containing solution into the hind paw of mice, determines the development of both an acute proprioceptive inflammatory pain component and a late phase based on persistent sensitization (wind-up) of spinal neurons ipsilateral to the peripheral lesion [24]. In this study, we showed that the subcutaneous injection of lebecetin, a C-type lectin isolated from *Macrovipera lebetina* snake venom, inhibited in a dose-dependent, biphasic hormetic-like way the nocifensive reactions and the neuroinflammation-associated cytokine expression alterations induced by formalin. The hormetic pharmacological response manifests itself as a dose/response relationship characterized by a biphasic effect. In fact, in the presence of various chemical stimuli, biological tissue can give opposite responses depending on the doses. This means that a true dose-dependent biphasic response is determined, so much so that the hormesis could also be interpreted as a complex adaptive response aimed at survival or maintenance of homeostasis [29, 30]. In general, the responses presenting a hormetic trend are characterized by a modest stimulation of a certain function at low doses and by its subsequent inhibition (*i.e*., toxic) at increasing or higher doses.

### 4.1 Effect of Lebecetin on Formalin-induced Nocifensive Behaviour

Intra-paw lebecetin reduced nociceptive responses (and the paw thickness) in the first and second phase of the formalin test with only the lowest doses, while the higher doses partially reduced the second nociceptive phase without any effect on the first one. These biphasic behavioral effects by themselves suggest a dose-dependent hormetic action of lebecetin. Previous studies have demonstrated that the targets of lebecetin (as well as of lebectin and lebecin, which are isoforms of lebecetin) are certain integrins (*i.e*. α5β1 and αv) which are inhibited by both isoforms, thus preventing cell adhesion, platelet activation, and tumor cell migration/invasion [15-18]. Consequently, lebectin, lebecin, and lebecetin have the same inhibitory integrin pattern, interfering with the adhesion to fibronectin and fibrinogen, the major ligands for α5β1 and αv integrins. The described mechanism of action of lebecetin may partly explain its biphasic effect in reducing pain more effectively at low doses than at higher doses. Some αvβ3 and αvβ5 integrin inhibitors (proposed as possible anti-angiogenic drugs for cancer treatment) have been reported to exhibit a paradoxical effect at nanomolar doses: a facilitatory effect of angiogenesis and tumor growth rather than inhibiting the tumor growth [31]. Low concentrations of these agents could modify the structure of integrins and, therefore, the trafficking of angiogenesis promoters (*i.e*., VEGF) and/or cell adhesion receptors, thus favoring cell migration and survival [31]. A similar biphasic effect was observed with alternagin-C, a cysteine rich disintegrin of *Rhinocerophis alternatus* venom, on cardiomyocyte physiology. Treatment with this biomolecule produced a hormetic dose-response curve, a powerful inotropic effect and enhanced cardiac pumping capacity function at low doses, while a modest inotropism at higher doses [32]. The current study extends the effect of lebecetin by showing its capability of reducing the formalin-induced nocifensive behavior, the associated skin edema, and the spinal cord morpho-functional sequelae. The observed hormetic-like effect of lebecetin may be also due to the bi-directional activation of the cell signaling of the integrin. It can occur both as binding with the extracellular matrix (ECM), (*i.e*., cell adhesion and migration) in response to intracellular signals or activation of intracellular signals in response to ECM binding [33] and in the specific case of nociceptive signals, it can be directly related to the stimulation of dorsal root ganglia (DRG) neurons [34, 35]. Based on this, the ECM components play a decisive role in the genesis and maintenance of inflammation, cell recruitment, and the subsequent peripheral nerve sensitization in the inflamed tissue by the recruitment and activation of specific integrins [36]. The action of lebecetin for pain relieving may be attributable to its ability to block α5β1 or αv integrins. By avoiding the activation of integrins, it can prevent the downstream activation of receptors for inflammatory mediators (*e.g*., prostaglandin and/or bradykinin receptors, TRP channels, *etc*.) associated with nociceptive behavior in the formalin test.

The inhibitory action of lebecetin on integrins can prevent a massive and long-lasting recruitment/enhancement of ion channel activation through the mobilization of intracellular signaling proteins (*e.g*., G-protein components or kinases) [37]. Furthermore, the inhibition of intracellular calcium mobilization by lebecetin and the consequent failure to activate intracellular signaling pathways, such as PKCε [38, 39] or upregulation of COX2, PGE2 and activation of the adenylyl cyclase/cAMP/PKA cascade should also be strongly considered [39-41]. Therefore, we believe that the inhibition of α5β1 and αv integrins by lebecetin may prevent a complex series of intracellular signals responsible for the inhibition of nociceptive behavior. Furthermore, we observed that lebecetin at a low dose was not capable of inducing painful behavior by itself, showing no observed adverse effect level, NOEL. This indicates that the α5β1 and αv integrins may not have a tonic control effect on the sensitization of the nociceptive nerve or their discharges, but only in an inflamed environment (*i.e*., in the presence of elevated inflammatory mediators or by the recall of inflammatory cells). It is conceivable that cyclases, kinases, and many other enzymes involved in the signaling transduction leading to inflammation and hyperalgesia cooperate within a complex ECM-integrin-cytoskeleton interaction to orchestrate a signaling hub [42]. It has been shown that the β1 integrin subunit plays a role in hyperalgesia regardless of the type of chronic pain [22]. This subunit is expressed in various cell types, including immune cells, that play a key role in the development and maintenance of chronic pain [43, 44]. Indeed, a necessary function of immune cells for the chronicization of both inflammatory and neuropathic pain is now well recognized [43]. The selective involvement of integrins in lymphocyte activation and motility has been demonstrated in neuro-inflammatory conditions. Thus, integrin β1 subunit-deficient T lymphocytes drastically lose their ability to spread to nerve tissue in models of experimental autoimmune encephalopathy (EAE) in rodents, and this effect is associated with the found, delayed or abolished development of active EAE [45].

### 4.2 Effect of Lebecetin on Formalin-induced Gene Expression

Formalin increased the expression of the cytokines IL-1β, IL-4, and TNF-α in the spinal cord one hour after the intrapaw injection. This suggests that in our model of formalin-induced inflammation, these cytokines, compared to the others analyzed here (*i.e.*, IL-6, IL-8, and IL-10),, participated more actively in the central maladaptive neuroplasticity involved in spontaneous pain and hyperalgesia induction. Furthermore, the fact that IL-4 expression is increased without a similar increase in IL-10, the other important anti-inflammatory cytokine, would suggest that IL-4 may assume a key compensatory function for local self-limiting neuroinflammation [46]. A predictably opposite trend was observed for gene expression of COX2 and iNOS, two key enzymes with a cellular protective function. Down-regulation of these two enzymes by intra-paw formalin (mainly by iNOS and partly by COX2) was observed in the spinal cord, and lower doses of lebecetin then reversed this effect. Consistently, the same recovery trend was found in serum for those enzymes. This indicates that although formalin damage is localized (*i.e*., paw), the change in the expression of cytokines, COX2, and iNOS affects different tissues or organs at a distance (spinal cord and blood). Thus, our data confirm that pathological pain is a systemic clinical condition rather than a problem confined exclusively to a single organ district. As observed with COX2 and iNOS, cytokine levels were also shifted towards more physiological values with lower doses of lebecetin. As mentioned above, such a dose-dependent hormetic-like effect of lebecetin was also observed in ameliorating pain or paw edema. Although our experiments do not allow us to understand why a greater pain-killer effect was observed at lower doses, here we may hypothesize different binding affinities on different integrins, *e.g*., α5β involved in the production of PGE2 [22], playing opposite roles in the induction of inflammatory cascades, or even the loss of selectivity at higher doses of lebecetin leading to the activation of other integrin receptors/pathways that can facilitate the inflammatory process and pain responses [47, 48]. In fact, it should be borne in mind that stimulation of lectin C receptors activates a complex immune response that models specific responses to any antigen [49]. It may be supposed that with increasing doses, lebecetin stimulates its own receptors (both membrane and cytosolic), which, by regulating NFkB in various ways (*i.e*., signaling through SyK and RAF1 to direct cytokine expression pattern), induce different phenotypes of antigen-presenting cells to differentiate, in turn, in several lymphocyte clones [50]. Thereafter, different clones of T lymphocytes produce various cytokines to organize a precise immune response. In this study, a gradual loss of the analgesic and anti-inflammatory effect with higher doses of lebecetin might be, therefore, conceivable following activation of the lectin receptors with consequent masking or occlusion of the protective effects by blocking the α5β1 and αv integrins with lower doses.

A confirmation that the blockade of those integrins may inhibit the synthesis of pro-inflammatory cytokines and instead increase the anti-inflammatory ones has been recently shown *in vitro* on macrophages derived from human monocytic THP1 cells exposed to LPS [21]. This multi-receptor activity of lebecetin could also explain why this biomolecule (at least at the best-protecting dose in this study) per se increased the expression of some inflammatory cytokines and reduced iNOS in healthy conditions. This apparently paradoxical defect may be linked to the very nature of a molecule that, by interfering with the immune system and its role in continuous functional adaptation, can exert pleiotropic effects, which are often opposite depending on a given pathophysiological context [51]. Thus, there is evidence that a well-studied C-type lectin receptor, Dectin-1 [52], depending on how different ligands in the surrounding environment may vary, may perform even opposite activities in immune regulation. In addition to pro-inflammatory responses, such as cytokine production and phagocytosis, tolerant responses have also been attributed to dectin-1 activation [53].

### 4.3 Effect of Lebecetin on Formalin-induced NS Neuron Hyperactivity

In this study, formalin caused a marked increase in the spontaneous activity, frequency, and duration of excitation of spinal NS neurons. Formalin also increased both the amplitude and slope of NS neurons. The amplitude and slope of APs correlate with synaptic strength, the magnitude of the synaptic currents and the number of activated input fibers of NS neurons [54]. Overall, the spinal sensitization following peripheral formalin administration that overlaps and underlies the nociceptive behavior has been widely reported [55-58]. What is noteworthy is the fact that even if lebecetin alone had no effect on the activity of spinal NS neurons, when administered before formalin and exactly 10 min before, it abolished the hyperactivity of spinal NS neurons, thus showing a neuroprotective effect. Intriguingly, the administration of lebecetin after the administration of formalin was able to normalize the NS neuron hyperactivation. The sensitization of spinal neurons is inevitably associated with the peripheral inflammatory cascade and neurochemical alterations induced by the peripheral administration of formalin, but even more, it is the expression of an altered local microenvironment, which, as described above, undergoes modifications in the expression of enzymes and cytokines involved in inflammatory processes. Therefore, the inhibitory effect of lebecetin on the spinal inflammatory microenvironment could also underlie the functional effects of NS neuron activity. Moreover, lebecetin showed effectiveness not only as a preventive treatment but also for overt neuroinflammation when administered after formalin. Although more in-depth experiments aimed at investigating the mechanisms underlying the effects of lebecetin on the activity of NS spinal neurons hyperactivated by formalin are required, we believe this preliminary data is interesting because it highlights a neuroprotective effect of lebecetin which may be useful in restoring neurofunctional alterations underlying chronicization of pain.

### 4.4 Effect of Lebecetin on Formalin-induced Microglia Activation

Finally, it has been recently demonstrated that spinal microglia contribute to sustained inflammatory pain *via* amplifying neuronal activity. In particular, spinal microglia activation seems to be associated with increased neuronal excitability. Indeed, the depletion of resident microglia cells reduced neuronal hyperactivity in the second phase of formalin-induced tonic pain [27]. Here, we confirmed the increased microglia proliferation in the superficial laminae of the dorsal horn of the spinal cord. Consistently, with the behavioral and functional data of electrophysiology, we found that the 0.32 nmol dose of lebecetin significantly reduced the microglia cell number in the same area. The microglial immune-driven component of the tonic phase of formalin also affected the infiltrating inflammatory cells in the hind paw skin, which were significantly reduced in microglia-depleted mice [27]. Consistently, we also found that the formalin-injected mice showed a dishomogeneous tissue in terms of dermal structures and collagen, together with an enhancement of infiltrating cells. These observational histological data also revealed that the dose of 0.32 nmol of lebecetin reduced both the morphological architecture impairments and the inflammatory reaction. Conversely, the higher doses of lebecetin worsened the tissue impairments induced by the formalin injection, assuming a hormetic/biphasic effect of this natural protein venom.

## CONCLUSION

This study further underlines the role of C-type lectins in the complex regulation of several cellular functions. This complexity is a consequence of the variable and extreme adaptability of their intracellular signaling activity under physiological or pathological conditions. In this study, we hypothesize that lebecetin, at higher doses, may act on the C-type lectin receptors (CLRs), which will be interesting to identify, while at lower doses, it more selectively inhibits some integrins (α5β1 and αv integrins). The outcome of the signals triggered by integrins and CLR, or their functional interaction, depends not only on these receptor systems but also on the nature or density of many other co-present ligands. Indeed, the microenvironment in which these protein/protein interactions occur plays a crucial role in one functional outcome rather than another. The inflammatory environment and the simultaneous presence of many other mediators at work is a very different condition from healthy tissue physiology. Therefore any biological or pharmacological alterations can also be very different. We can therefore imagine for lebecetin, and more generally for lectins, that they can have a versatile action and perform a multitude of functional adaptations to preserve the functional integrity of tissues and, in general, to maintain cellular homeostasis. Consequently, the protein-protein interaction between lebecetin and CSF and/or integrins within the extracellular matrix may be critical in shutting down the signaling systems that mediate inflammation and hyperalgesia. These findings further encourage the search for new targets and improved associated therapies against inflammatory pain.

## Data Availability

Not applicable.

## LIST OF ABBREVIATIONS

CLRs: C-type Lectin Receptors
DRG: Dorsal Root Ganglia
eAPs: Extracellular Action Potentials
ECM: Extracellular Matrix
NS: Nociceptive-specific
