# Supplementary material for: Biphasic Hormetic-like Effect of Lebecetin, a C-type Lectin of Snake Venom, on Formalin-induced Inflammation in Mice
Source: Curr Neuropharmacol. 2023 Dec 8;22(8):1391–405. doi: 10.2174/1570159X22999231207105743 (PMC11092918; doi:10.2174/1570159X22999231207105743)
Supplement: Supplementary file 1 — Supplementary material is available on the publisher’s website along with the published article. [file CN-22-1391_SD1.pdf]

Supplementary Material

Biphasic Hormetic-like Effect of Lebecetin, a C-type Lectin of Snake Venom, on Formalin-induced Inflammation in Mice

Carmela Belardo[1,#], Jed Jebali[2,#], Serena Boccella[1,#], Rosmara Infantino[1], Antimo Fusco[1], Michela Perrone[1], Roozbe Bonsale[1], Iolanda Manzo[1], Monica Iannotta[1], Damiana Scuteri[3,*], Franca Ferraraccio[4], Iacopo Panarese[4], Giovanna Ferrara[4], Francesca Guida[1], Livio Luongo[1], Enza Palazzo[1], Najet Srairi-Abid[2], Naziha Marrakchi[2] and Sabatino Maione[1,*]

[1]Department of Experimental Medicine, Pharmacology Division, University of Campania "L. Vanvitelli", Naples, Italy; [2]Laboratory of Biomolecules, Venoms and Theranostic Applications, LR20IPT01, Institut Pasteur of Tunis, University of Tunis El Manar, Tunis 1002, Tunisia; [3]Pharmacotechnology Documentation and Transfer Unit, Preclinical and Translational Pharmacology, Department of Pharmacy, Health and Nutritional Sciences, University of Calabria, Rende, Italy; [4]Pathology Unit, Department of Mental and Physical Health and Preventive Medicine, University of Campania "L. Vanvitelli", Naples, Italy

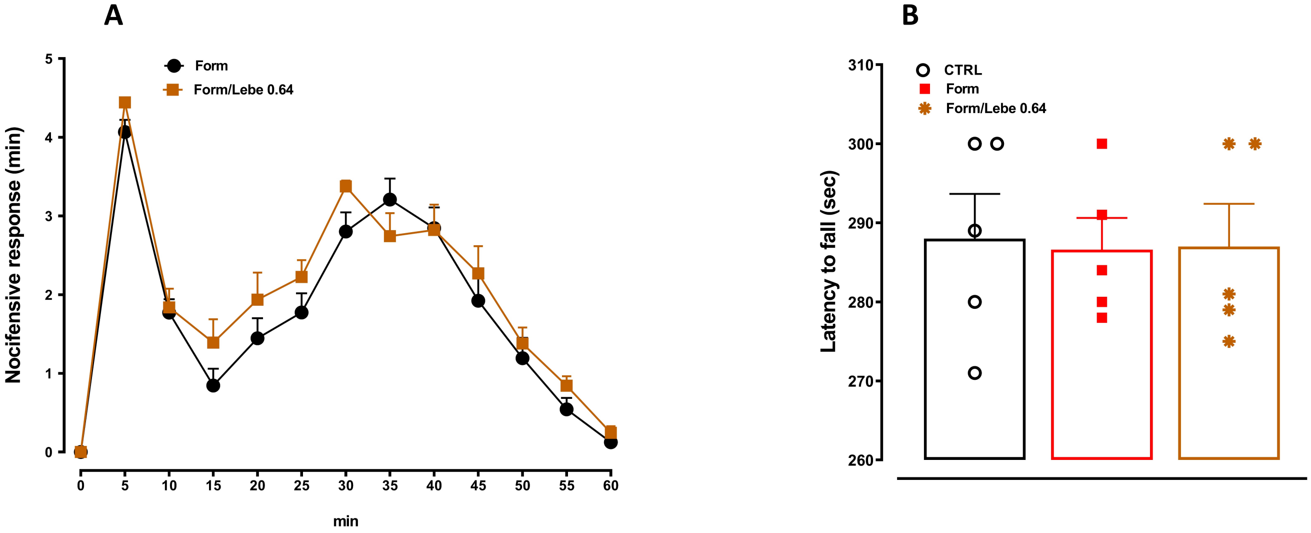

Fig. (S1). (**A**) Effect of the systemic administration of vehicle (PBS, 1%) or lebecetin (Lebe, 0.64 nmol, s.c.) on the nocifensive response in the formalin test. (**B**) Effect of the systemic administration of vehicle (PBS, 1%) or lebecetin (Lebe, 0.64 nmol, s.c.) in mice that received formalin 1.25% in the dorsal surface of the hind paw on the latency to fall (sec) in the rotarod test (Deli, S.B.; Bonab, S.I.; Khakpay, R.; Khakpai, F.; Feyzi, M.H. An interaction between basolateral amygdala orexinergic and endocannabinoid systems in inducing anti-nociception in the rat formalin test. *Psychopharmacology (Berl),* **2022**, *239(10):3171-3184*). The motor coordination was also evaluated in naïve mice (CTRL). Data are represented as mean ± SEM of 5 mice per group. $P < 0.05$ was considered statistically significant, two-way ANOVA followed by Dunnet's post-hoc test.

1875-6190/24 $65.00+.00 © 2024 Bentham Science Publishers
